# Human pericentromeric tandemly repeated DNA is transcribed at the end of oocyte maturation and is associated with membraneless mitochondria-associated structures

**DOI:** 10.1038/s41598-020-76628-8

**Published:** 2020-11-12

**Authors:** M. A. Dobrynin, N. M. Korchagina, A. D. Prjibelski, D. Shafranskaya, D. I. Ostromyshenskii, K. Shunkina, I. Stepanova, A. V. Kotova, O. I. Podgornaya, N. I. Enukashvily

**Affiliations:** 1 Institute of Cytology RAS, Saint Petersburg, Russia; 2 Ava-Peter – Scandinavia Assisted Reproductive Technology Clinic, Saint Petersburg, Russia; 3 Center for Algorithmic Biotechnology, St. Petersburg State University, Saint Petersburg, Russia; 4 Faculty of Biology, St. Petersburg State University, Saint Petersburg, Russia; 5 North-Western State Medical University Named After I.I. Mechnikov, Saint Petersburg, Russia

**Keywords:** Germline development, Long non-coding RNAs

## Abstract

Most of the human genome is non-coding. However, some of the non-coding part is transcriptionally active. In humans, the tandemly repeated (TR) pericentromeric non-coding DNA—human satellites 2 and 3 (HS2, HS3)—are transcribed in somatic cells. These transcripts are also found in pre- and post-implantation embryos. The aim of this study was to analyze HS2/HS3 transcription and cellular localization of transcripts in human maturating oocytes. The maternal HS2/HS3 TR transcripts transcribed from both strands were accumulated in the ooplasm in GV-MI oocytes as shown by DNA–RNA FISH (fluorescence in-situ hybridization). The transcripts’ content was higher in GV oocytes than in somatic cumulus cells according to real-time PCR. Using bioinformatics analysis, we demonstrated the presence of polyadenylated HS2 and HS3 RNAs in datasets of GV and MII oocyte transcriptomes. The transcripts shared a high degree of homology with HS2, HS3 transcripts previously observed in cancer cells. The HS2/HS3 transcripts were revealed by a combination of FISH and immunocytochemical staining within membraneless RNP structures that contained DEAD-box helicases DDX5 and DDX4. The RNP structures were closely associated with mitochondria, and are therefore similar to membraneless bodies described previously only in oogonia. These membraneless structures may be a site for spatial sequestration of RNAs and proteins in both maturating oocytes and cancer cells.

## Introduction

Large-scale genome surveys suggest that most of the human genome is non-coding. One of the non-coding DNA families is tandemly repeated DNA (TR DNA), which constitutes approximately 10% of the human genome^1^. Tandemly repeated DNA is organized as multiple copies of a homologous DNA sequence of a certain size (repeat unit or monomer). The copies are arranged in a head-to-tail manner to form tandem arrays. The tandem repeats are classified in three major classes based on the size of the repeated sequence: ‘microsatellites’ for short repeat units (usually < 10 bp), ‘minisatellites’ for head-to-tail tandem repeats of longer units (> 10 and < 100 bp), and ‘satellites’ for even larger units (> 100 bp)^1^. In humans, the classical, or big, satellites 1, 2 and 3 (human satellites, HS) have been localized to pericentromeric regions. These pericentromere satellites belong to human satellite 1, 2 and 3 (HS1, HS2, HS3) families^2,3^. HS2 and HS3 are closely related; both are based on an ATTCC repeat. However, HS2 and HS3 sequences and organization are clearly distinct. HS3 shows a strict periodicity of 5 bp, and HS2 is built from two related units of 23 and 26 bp^4^. A number of chromosome-specific HS2 and HS3 families have been reported. Cross hybridization often occurs due to high similarity of the sequences^5^. Sometimes HS2 and HS3 are referred to as the HS2/3 family^6^.

At the end of the last century, the first data appeared on the role of big satellites. Their transcription was shown in different organisms: mice^7^, amphibians^8^, fish^9^, birds^10,11^ and humans^12,13^. The number of HS transcripts increases many fold at the beginning of proliferation, cell aging, cell differentiation, carcinogenesis, and cell stress^12–17^. In mouse and human embryos, the transcription of pericentromeric satellites was demonstrated at the 2- and 4-cell stages stages^7,18^. In human 4-cell embryos, about 20% of sequence raw reads (SRR) are satellite reads^18^. Transcription is then slowly downregulated until the blastocyst stage. However, the functional significance of the transcription is established only in mice, where pericentromeric satellite transcripts play an important role in embryogenesis. The transcripts are involved in the formation of heterochromatin association regions—chromocenters. Blocking the transcription leads to impaired formation of chromocenters and embryo development arrest at cell stages 2–4^7,19^. It was previously believed that all repetitive DNA is transcribed only in embryogenesis but not in germ cells. Once some oocyte transcriptomes had been analyzed, however, this assumption was revised. The non-coding repeated DNAs of dispersed repeats (transposable elements) play a key role in determining oocyte-specific transcription events^20^. TR DNA transcription in GV–MII oocytes is not established in human Recently, some datasets were published that allow study of total non-coding satellite transcripts of the human maturating germinal vesicle (GV), metaphase I (MI) and metaphase II (MII) oocytes of healthy donors^21–23^.

In oocytes, transcriptional activity is high during a growth phase, when chromatin remains in an ‘open’ conformation. The growing stage is succeeded by a phase of resumed meiotic activity and transcriptional silencing. Meiotic resumption is triggered in fully grown late GV oocytes before the germinal vesicle (i.e., the swollen nucleus of an oocyte before its disassembly) breakdown (GVBD) stage in response to the luteinizing hormone surge, initiating chromatin condensation, completion of meiosis I (with extrusion of the first polar body), and progression to metaphase of meiosis II before ovulation. At fertilization, the oocyte completes the final stages of meiosis II (extruding the second polar body)^24^.

Human GV oocytes stop at the diplotene prophase 1 stage of meiosis. At this stage, large-scale changes occur in the oocyte chromatin configuration and nucleoli organization. The conversion of the reticular nucleoli of oocytes to nucleolus-like bodies (NLBs) occurs at this time^25^. Chromatin initially forms multiple compact chromocenters associated with NLBs. This configuration is called a ‘non-surrounded nucleolus’ (NSN)^26^. NSN oocytes are transcriptionally active and synthesize all classes of RNA. The NSN configuration is succeeded by an intermediate configuration of 'partly surrounded nucleolus'. At the end of the GV stage, chromatin condenses and forms a rim of heterochromatin surrounding nucleolus-like bodies, acquiring a configuration termed ‘surrounded nucleolus’ (SN)^26^. The transition to SN is characterized by a progressive increase in condensation, transcriptional silence, and global DNA methylation^26–30^. The transcription arrest at the end of GV stage is accompanied by chromatin reorganization. The result of the genome reorganization is called the karyosphere^27^. GVBD commences at the onset of nucleolar shrinkage, with chromosomes assembled around the shrinking nucleolus. Shortly after nucleolar breakdown, the nuclear membrane starts to break down, and a maturating oocyte proceeds to the next stages of meiosis.

Studying the role of TR DNA in human oogenesis raises ethical and methodological difficulties. However, the late preovulatory GV, germinal vesicle breakdown (GVBD), MI, and (more rarely) MII oocytes are accessible for research.

The aim of this study was to analyze HS2 and HS3 transcription, and cellular localization of transcripts, in human maturating oocytes.

Here we report for the first time that, during oocyte maturation, the maternal transcripts of HS2 and HS3 are detectable at the late GV–GVBD stage, and are deposited in the ooplasm during the transition to the MI stage. The transcripts are polyadenylated and are present in published datasets of MI and MII oocytes. Using pericentromere oligonucleotide probes, we revealed the transcripts in membraneless coacervate bodies that contained helicases DDX4 and DDX5, and were adjacent to mitochondria. These features are a hallmark of membraneless, mitochondria-associated, nuage granules observed previously only in oogonia.

## Results

### Localization of pericentromeric HS3

**Figure 1:**
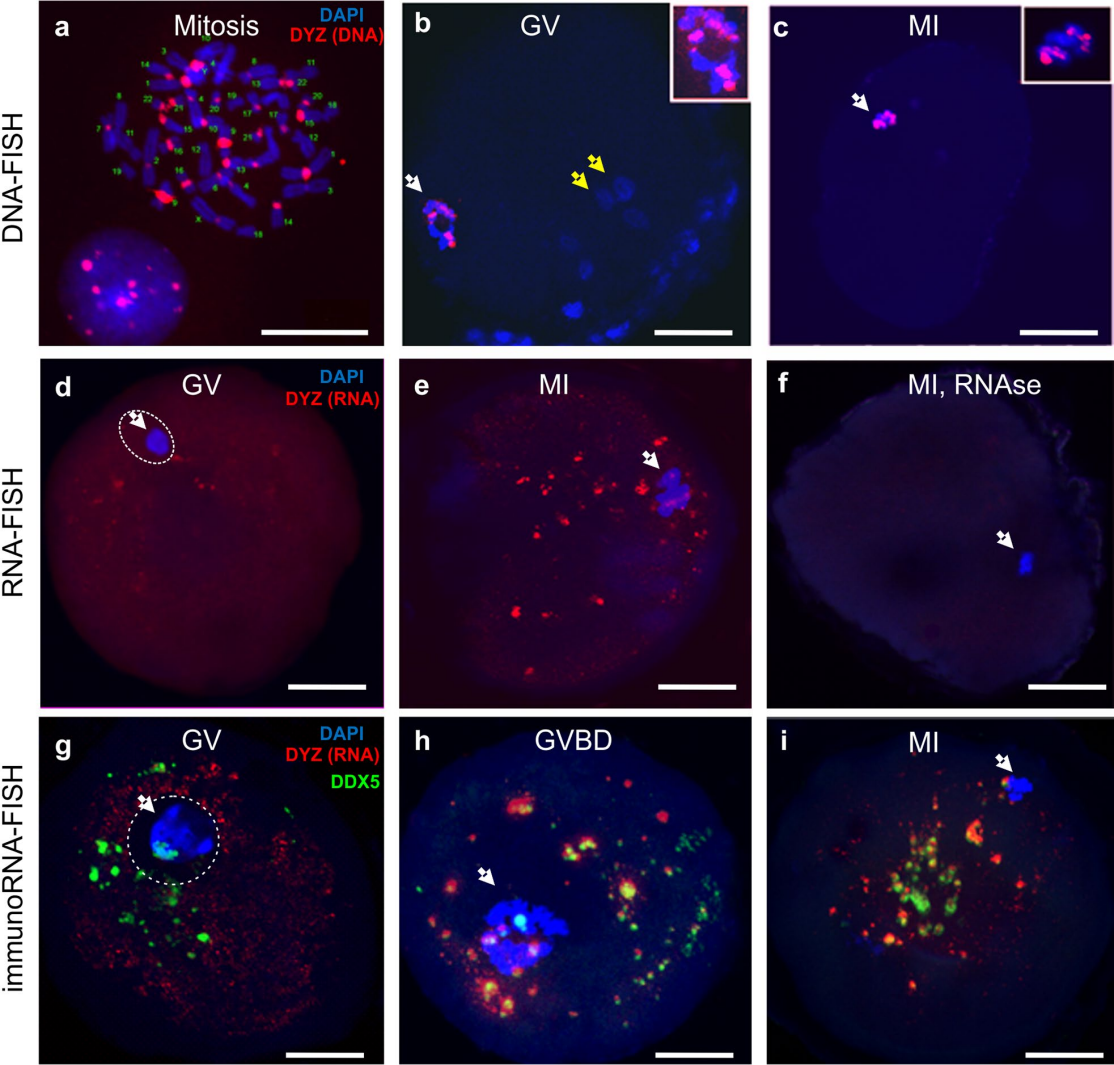
Spatial distribution of HS2/HS3 DNA (**a**–**c**), RNA (**d**–**f**), and a helicase, DDX5, relative to HS2/HS3 RNA (**g**–**i**) in human germinal vesicle (GV) and metaphase I (MI) oocytes. (**a**–**c**) Localization of HS2/HS3 DNA probed with DYZ1 (red) in metaphase spreads of human lymphocytes (**a**), in human GV (**b**) and MI (**c**) oocytes as revealed by DNA–DNA FISH; (**d**–**f**) localization of HS2/HS3 RNA probed with DYZ1 (red) in human GV (**d**) and MI (**e**,**f**) oocytes, as revealed by DNA–RNA FISH without (**d**,**e**) and with (**f**) RNase A treatment; (**g**–**i**) localization of the HS3 transcript probed with DYZ1 (red) and DDX5 RNA helicase (green) in GV oocytes (**g**); during the transition from GV to MI (**h**); and at the MI stage (**i**). White arrows—(**b**,**d**,**g**) a ring of highly condensed heterochromatin, (**c**,**e**,**f**,**h**,**i**) a metaphase plate. Yellow arrows—(**b**) cumulus cells. Inserts show (**b**) the heterochromatin ring and (**c**) the metaphase plate. The nucleus was encircled with dotted white line (**d**,**g**) after the merge with brightfield data (not included in the panel). Chromatin was counterstained with DAPI (blue). Scale bars: (5 μm) (A) and (50 μm) (**b**–**i**).

TR DNA families of big satellites are chromosome specific. The DYZ1 probe is a fragment of the HS arrays on the Y chromosome, but bioinformatics analysis showed that this DNA sequence was present in most chromosomes (see “Materials and methods” section). DNA–DNA fluorescence in-situ hybridization (FISH) confirmed these data. In the conditions described in in “Materials and methods’, the probe hybridized to the pericentromeric regions of most chromosomes (Fig. 1a). Thus, the probe could be used to identify a wide spectrum of HS2 and HS3 DNA arrays and transcripts originated from different chromosomes, and was therefore employed as a probe for subsequent experiments.

In human preovulatory GV oocytes, the hybridization signal was detected as part of a highly condensed heterochromatin ring in DNA–DNA FISH experiments (Fig. 1b). Thus, at this stage, the pericentromeric regions of the chromosomes were a part of the heterochromatin ring surrounding the NLB (nucleolus-like body), and were not detected in other regions of the oocyte. At the MI stage (Fig. 1c), the HS2/HS3 DNA was detected only on condensed metaphase chromosomes, but not in any extrachromosomal structures.

Thus, in a preovulatory oocyte, HS2 and HS3 DNA is located exclusively in the nucleus or chromosomes. The DYZ1 probe did not hybridize to any granule outside the nucleus (GV) or chromosomes (MI) in the ooplasm in the DNA–DNA FISH conditions we used (see “Materials and methods”).

### Localization of pericentromeric HS2/HS3 transcripts

DNA–RNA FISH was performed in order to study the localization of the HS2 and HS3 transcripts in GV and MI oocytes. At the GV stage (Fig. 1d), the HS2/HS3 transcript was detected in the oocyte cytoplasm as small granules. At the MI stage (Fig. 1e), the number of punctate foci increased. The granule size varied from 200–500 nm to 3–4 μm. No signal was detected in cells treated with RNase A (Fig. 1f). Thus, HS2 and HS3 transcripts existed in maturating oocytes. Transcripts were detectable in oocytes at the GV stage. However, the number of relatively large (more than 200 nm) HS2/HS3 foci increased at MI. These data suggest the transcription occurred at the end of GV before global genome silencing.

### Colocalization of pericentromeric HS2 and HS3 transcripts, DDX5 and DDX4 RNA helicases and mitochondria

At the end of MI stage, HS2 and HS3 RNAs were revealed as granules varying in size. Germ cells of various organisms are characterized by special cytoplasmic RNP granules that are commonly called germ granules^31,32^. One of the membraneless germ granules described to date in mammalian female germ cells are nuage ooplasm granules. Nuage granules associated with mitochondria form a structure that in Mammalia can be detected only in oogonia—Balbiani bodies^33^. Nuage structures contain some helicases, and the RNA-helicase DDX4 (MVH, human VASA) is established as a hallmark of germ granules in female early germ cells, oogonia^34^. In some other germ granules, such as the chromatoid body, a male germ granule, other helicases have been revealed, including DDX5, that often colocalize with repressed mRNA and are capable of binding HS2 and HS3 DNA^32,35^. Therefore, in the next step, we examined the spatial association of HS2/HS3 transcripts, helicases DDX5 and DDX4, and mitochondria by combination of FISH and immunostaining with the antibodies (ABs) against DDX4, DDX5 and the AB against import receptors Tom20 of mitochondrial preprotein translocases of the outer membrane.

**Figure 2:**
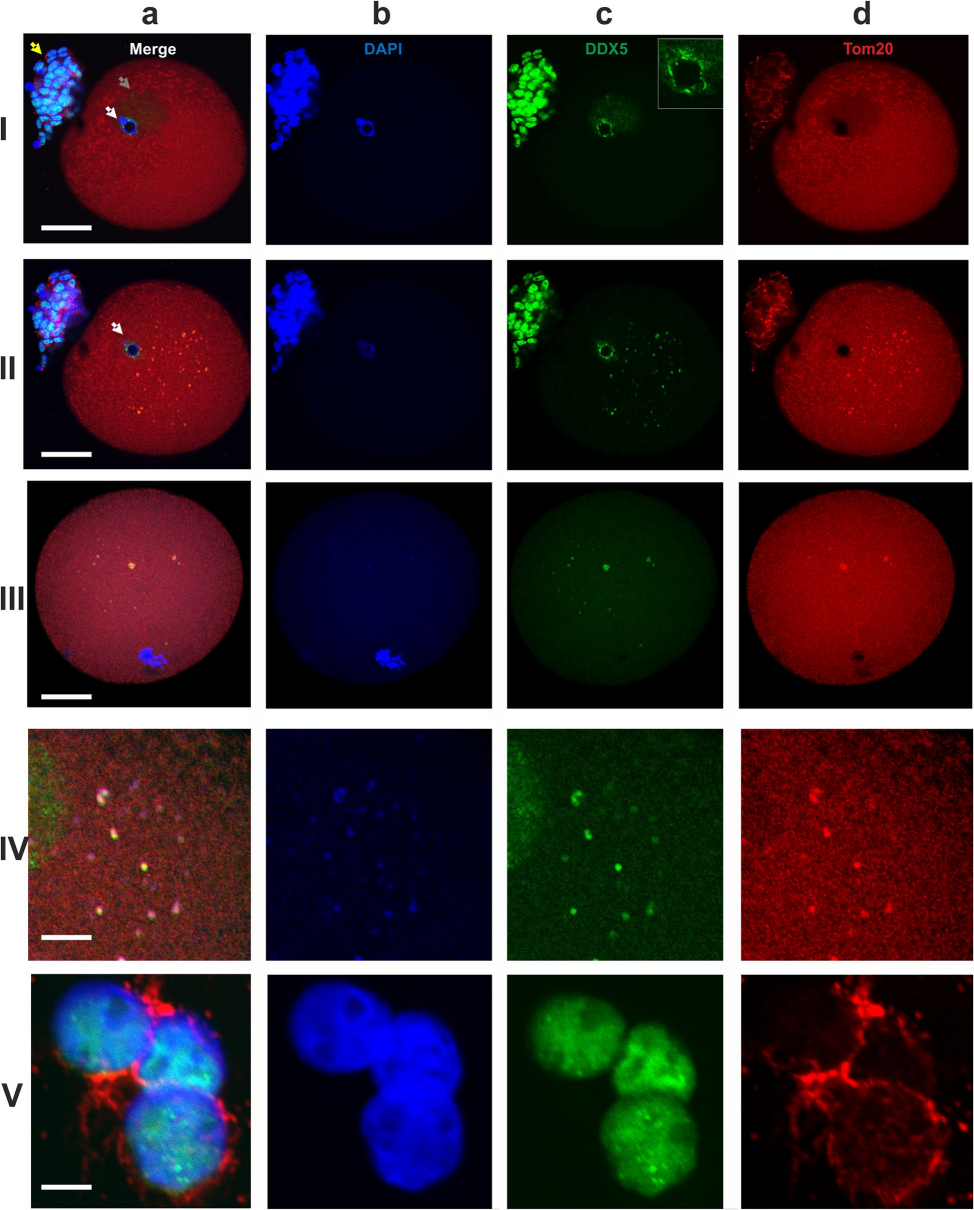
Localization of the Tom20 (**d**, red) and DDX5 RNA helicase (**c**, green) in two different optical sections of a GV oocyte (I—intranuclear, II—ooplasmic DDX5), in an optical section of an MI oocyte (III) and in cumulus cells (V). The ooplasmic structures co-stained with the AB against Tom20 and DDX5 are shown at a higher magnification in (IV). The insert in the image 2-Ic shows a magnified area of heterochromatic ring stained with the antiDDX5 AB. White arrows—a ring of the highly condensed heterochromatin; gray arrow—the nucleus of a GV oocyte; yellow arrows—residual cumulus cells. Chromatin is counterstained with DAPI (**b**, blue). The pseudocolored merged images are shown in (**a**). Scale bars—50 μm (I–III), 10 μm (IV–V).

At the GV stage, very small hybridization signals from the transcripts were detected in the ooplasm (Fig. 1g). The antiDDX5 AB revealed the protein in the ooplasm (Figs. 1g, 2-IIc) as well as adjacent to the heterochromatic ring DNA in the nucleus (Fig. 2-Ic). In some oocytes, the antiDDX5 AB stained the nucleus diffusely (Figs. 2-Ic, 3a) while in others it stains in a punctate manner (Fig. 1g). Colocalization of HS2/HS3 RNA and DDX5 was not observed in the nucleus (Fig. 1g). The intranuclear DDX5 was adjacent to the heterochromatic ring surrounding the NLB (Fig. 2-I, II). However, partial overlapping occurred in the ooplasm (Fig. 1h,i). The major part of the cytoplasmic pool of the protein was revealed as brightly stained granules that overlapped with some mitochondria signals (Fig. 2-IVb). The granules were weakly stained with DAPI (Fig. 2-Ib), a dye capable of weak interaction with RNA^36^. In contrast, stained granules were rarely observed in RNase A-treated cells, suggesting that RNA was indeed responsible for the weak staining observed in DAPI-stained cells untreated with RNase (e.g. Fig. 1f). However, the staining can at least partially correspond to mitochondrial DNA.

During the transition from the GV to MI stage, the HS2/HS3 transcripts were detected as granules in the ooplasm and near the chromosomes of the forming metaphase plate (Fig. 1h). DDX5 was observed in the ooplasm and near the chromosomes. The diameter of stained granules was under 2 µm. HS2/HS3 RNA and DDX5 ooplasmic granules were often colocalized.

At MI, the HS2/HS3 transcripts were detected mostly in ooplasm; little or no signal was observed near the chromosomes (Fig. 1e,i). The diameter of the hybridization signals was up to 2 µm. The AB revealed brightly stained spots (also up to 2 µm) in the ooplasm, with some minor spots adjacent to chromosomes of the metaphase plate. HS2/HS3 RNA and DDX5 ooplasmic granules were often colocalized, as indicated in Fig. 1. The antiDDX5 staining partially overlapped with mitochondria revealed by antiTom-20 staining (Fig. 2-III, IV).

The overlapping of the antiDDX5 and Tom20 immunostaining was not observed in cumulus cells (Fig. 2-V) and, thus, it is unlikely to be an artifact of the co-staining procedure. The difference between staining patterns in somatic cumulus cells and late oocytes was dramatic: in late GV and in MI, the HS2/HS3 transcripts were colocalized with helicase DDX5 granules. The latter overlapped with some of the ooplasm mitochondria. In adjacent cumulus cells, overlapping of the helicase and mitochondria never occurred in our experiments.

Thus, in late GV–MI and in MI, the HS2/HS3 transcripts were colocalized with a helicase, DDX5, located in granules that overlapped with some of the oocyte mitochondria.

**Figure 3:**
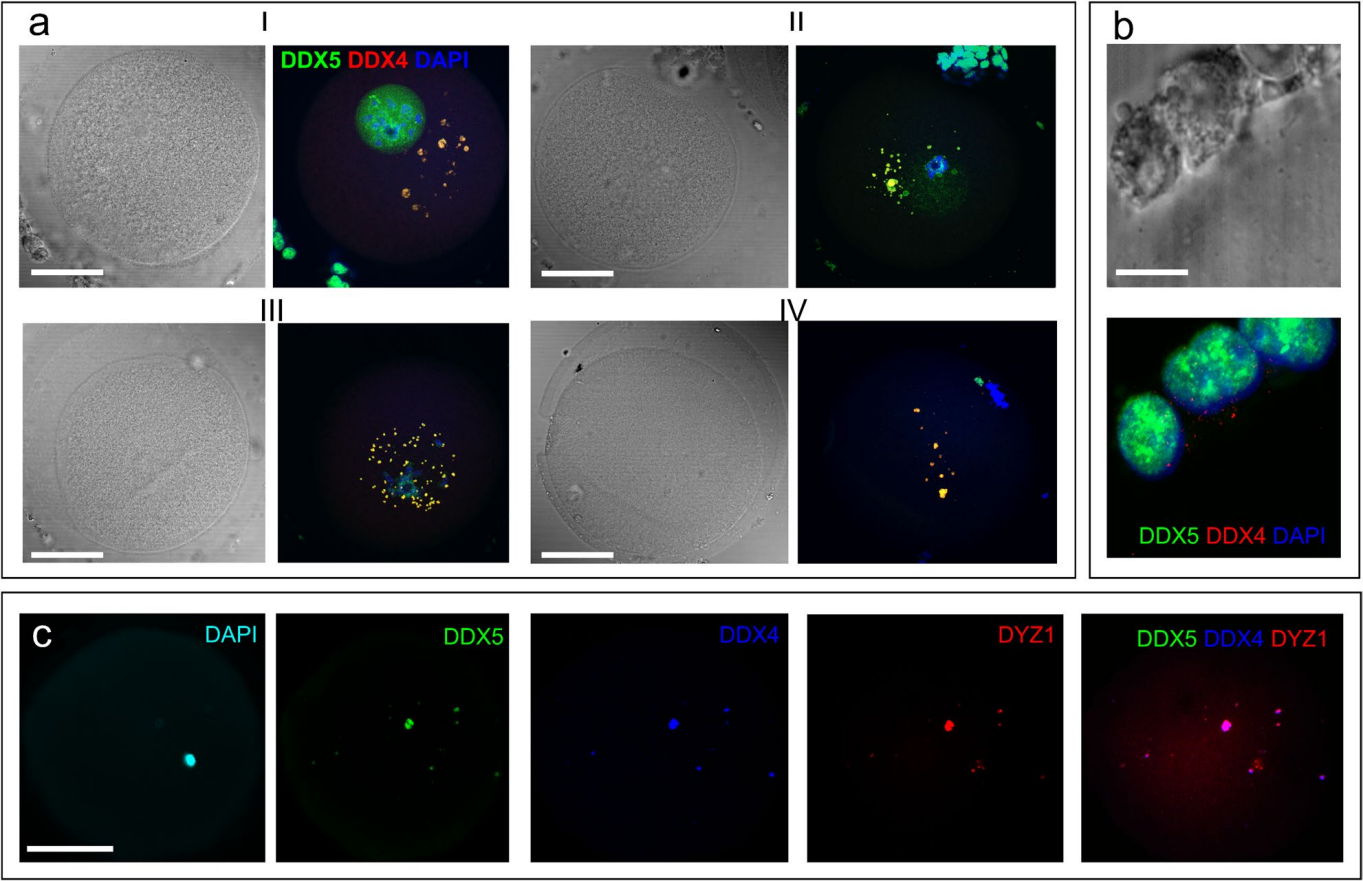
Localization of the DDX5 (**a**–**c**, green) and DDX4 (**a**,**b**—red, **c**—blue) RNA helicases in GV (**a**, I), late GV (**a**, II and **c**), GVBD (**a**, III) and MI (**a**, IV) oocytes) and in cumulus cells (**b**). Chromatin is counterstained with DAPI (**a**,**b**, blue; **c**—cyan). Pseudocolored merged images of a single optical section are shown in (**a**), (**b**). Black and white images in (**a**), (**b**) represent phase contrast images taken simultaneously with the corresponding optical section. The overlapping of helicases with DYZ1 DNA–RNA FISH signals is shown in c. Scale bars—50 μm (**a**,**c**), 10 μm (**b**).

The DDX5-containing cytoplasmic granules were co-stained with the antiDDX4 AB in oocytes, but not in cumulus cells (Fig. 3a,b) where DDX5 was revealed only in the nucleus (Figs. 2-V, 3b). Most of the granules stained with the antiDDX5 ABs contained HS2/HS3 transcripts (Fig. 3c).

### HS2/HS3 transcripts from both chains are present in human preovulatory oocytes

The DYZ1 probe is built of ATTCC repeats. However, unlike the coding DNA, non-coding DNA can be transcribed from both chains. In heat shock, the GGAAT chain is predominantly expressed, while in senescent fibroblasts and some cancer lines, the transcribed chain is ATTCC^12,16^. In the experiments described above, we demonstrated the transcription of ATTCC HS2/HS3 DNA chain. The questions, whether the transcription occurred also from the GGAAT chain and where the transcripts were localized, were addressed in double RNA—FISH experiments with the DYZ1 probe and a probe, PCT14, designed on the basis of the GGAAT repeat and its variations. The PCT14 probe originated from the chromosome 14 TR DNA but it also hybridized to pericentromeric regions of other chromosomes (Supplementary, Fig S1a). The hybridization signals obtained from the PCT14 probe overlapped with those obtained from the DYZ1 probe. However, in GV oocytes, the majority of the DYZ1 hybridization sites were free from the green PCT14 signals, while all the green signals colocalized with the DYZ1 (Supplementary, Fig S1b). In MI, the PCT14 and DYZ1 hybridization sites were completely overlapping (Supplementary, Fig S1c).

The results of double RNA-FISH confirmed that HS2/HS3 transcription occurred from both chains. The patterns of hybridization signals are slightly different in GV and MII. In GV oocytes, the DYZ1 signal prevailed, while PCT14 was less prominent. In MI oocytes, DYZ1 and PCT14 signals were almost completely overlapping. Therefore, the GGAAT strand transcription is probably delayed. A difference in PCT14 and DYZ1 hybridization raises the possibility of different timing in transcriptions from ATTCC and GGAAT strands.

### Quantification of HS2/HS3 transcription in human oocytes and cumulus cells.

In FISH experiments, HS2/HS3 transcription took place at the late GV–GVBD stages (Fig. 1), but little or no RNA-FISH signals were detected in cumulus cells (Supplementary, Fig. S2a, b). The quantities of HS2/HS3 transcripts in cumulus cells and GV were compared using real-time PCR (Supplementary, Fig. S2c). Two genes were used as references: GAPdH and β-actin. GAPdH is the most widely used reference gene, however a maturing oocyte is a highly specialized cell, where many genes act in a different manner than in somatic cells. The non-stable transcription of GAPdH during bovine oogenesis especially during the GV–MII period, when its level is tenfold downregulated, was demonstrated^37^. However, GAPdH is considered to be one of the most stable references for porcine maturating oocytes^38^. Therefore we decided to use both GAPdH and β-actin references.

In both lines of experiments, the level of HS2/HS3 transcription was many fold higher in oocytes as compared to cumulus cells. However, the fold change value was different depending on the reference gene used for calculations. In our experiments, GAPdH average threshold cycles were 27.2 ± 0.4 for cumulus cells and 36.7 ± 0.75 for oocyte samples. Probably, in human oogenesis, its transcription is downregulated as has been shown for bovine oogenesis. The β-actin threshold cycles were relatively stable: 28.2 ± 0.3 in oocytes versus 26.5 ± 1.1 in cumulus. Thus, we consider the data calculated with the β-actin reference gene to be more reliable.

### HS2/HS3 sequences share homology with the DYZ1 probe in human preovulatory oocyte transcriptomes

We have revealed HS2/HS3 transcripts in situ using the DYZ1 probe. In the next step, we searched the oocyte transcriptomes for sequences that shared a homology with the DYZ1 probe. Several human preovulatory oocyte transcriptomes have been published^21–23^. We analyzed the original sequence raw read (SRR) datasets, as described in the “Materials and methods” section. To avoid inclusion of DNA contamination sequences we analyzed only polyadenylated sequences—since both trimming and assembly software remove poly-A/T tails, initial untrimmed reads were mapped back to these transcripts^39^ to check for the presence of poly-A tails. As a result, only polyadenylated transcripts from different datasets were used for calculations. Their length ranged from 300 to 640 bp. A representative consensus sequence was computed for each cluster and further quantified using Kallisto^40^ (Supplementary Table 1). The most abundant of them are shown in Table 1. Among these transcripts, the transcript 1 from SRR5295901 has the highest TPM (transcripts per million) value and comprised majority of total TPM for transcripts shown in Table 1. In addition, we estimated the total expression of three known reference HS2/3 sequences to check whether the quantity trends for clusters’ consensuses corresponded to trends for already-known sequences. In all transcriptomes shown in Supplementary Table 1, the reference sequences were detected. The clusters’ TPM changed from one SRR to another in the same manner as the reference TPM.

**Table 1:** Analysis of published human MI/MII oocytes transcriptomes: list of the most abundant HS2/HS3 transcripts that share homology with the DYZ1 probe.

Publication	Accession no. of dataset	Transcript
Reyes et al.^22^	SRR5295893	TTTTTTTTTTTTTTTATTCCATTCAAGTCCATTCCATTCCATTCCATTCTATTCCATTCCATTCCACTCCGCTCCAATCCATTCCATTCGAGCCCATTCCATTCCATTATATTCCATTCGACTCTATTCCGTTCCAATACTCTTGAGTCCATTCCATTCCACTCCATTCCAATCCATTACATTCCTTTCGAGTCCATTCCATTGCTTCCAAATCCTTTCCATTCAATTTCATTCGAGTCCATTCCATTCCACTCCATTCTATTCCATTCAAGTCCATTCCTTTGCATTCCACTCCATTCCATTCCATTCCATTCCAGTCCATTTCATTTCATTGCATTTCATCCCAGTCCATTCCATTCCACTTCTTTCCATTCCATTCATGTCCATTCAATTCCATTCCATTCGTGTACATTCC
	SRR5295901	Transcript 1 TTTTTTTTTTTTTTTTTTTTTTTTTTTCATTCCATTCCATTCCATTCCATTCCATTCCATTCCATTCCATTCCATTGCATTCCATTCCATTCCATTCCATTGCACTGCACTCCATTCCATTACATTCTACTCTATCTGAGTCGGTTTTATTGCATTAGATTCTATTCCATTGGATTACTTTCCATTCGATTACATTCCATTCATGTACATTCCATTCCAGTCAATTACATTCGAGTTCATTACGTTACATTCCAGTATATTCCATTGTATTCGATCCCATTCCTTTCAATTCCATTTCATTCGACTCCATTATATTCGATTCCATTCCACTCGAATCCATTCCATTAGAGGACATTCCATTCAGAT Transcript 2 TTTTTTTTTTTTTTTTTTTTTTTTTTTTTGCCATTCTATTTCATTGCAATGCATTCCATTCCATTCCATTCCATTCCATTCCATTCCATCACATTACATTACGTTTGCACCCAGTTCATTTCATGCCATTCGATGCCAATACATACCATTCCATTCGAATCCATTCAATTTCATTCCATTATAGTCAATTCCATTCCATTCAATTATATTACATTCCTTTCAAGTCTATTCCATTCCATTACTTTCCATTTGTGACCATTCCATTGCATTCCATTCGAGTCCATCCACTCGAGTCCATTCCATTCCATTCCATTCCATTCCTTTCGAGTCCACTCAATTCCATAG
Zhang et al.^23^	SRR6350575	TTTTTTTTTTTTTTTTTTTTTTTTTTTTTCGATGATTGTTCCATTCGATTCTGTTCGGTGATTCCATTCGATTCCATTTGATAATGATTCCGTTCGAGACCATTCGATGATTCCATTCAATTCCATTCAATAAAGATTCCATTCGAGTCCATTCATTGATTCCCTTCAAGTCCATTCGATGATTCCATCAGATTCCATTCAATGAATCCATTCGATTCCATTCAGTGATGATTCCATTCATTTCCATCTGATGATGATTCCATTCGATTCCATTCAATGATTCCATTCAATTCCATTTGATGATGATTTCAATCAATTTCATTCGGTGATTCCATTCGAATCCACTCGATGATGAGTCCATCCATTTCAATTTCATGATAATTCCATTCGTTTCCTTTCGATGGCGTTTCCATTCGATTCCATTCGATGTTGATTCCATTTGTTTCCATTGGATGATGATTCCGTTCGTGTCCATTCGATGATGATCATATTGGATTTCATTCCATAATTCTATTCGAATCCATTTGATGAT

**Figure 4:**
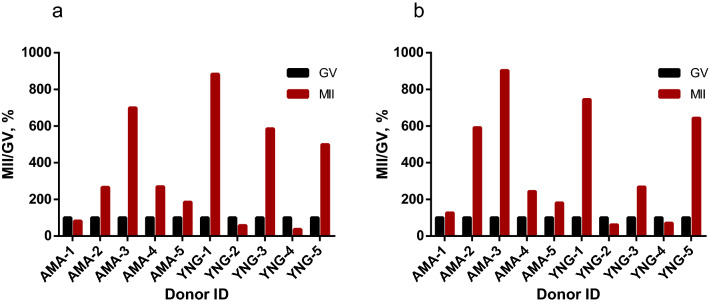
Computational analysis of HS2/HS3 transcripts quantity in GV and MII individual oocyte transcriptomes published by Reyes et al.^22^. (**a**) TPM calculated for transcripts clustered using UCLUST (see “Materials and methods”); (**b**) Total TPM of three already-known HS2/HS3 sequences: X60726.1, S90110.1, X82942.1. The already-known sequences were taken as references to compare their TPM value with the TPM value of clustered transcripts shown in (**a**). X-axis, donor ID as shown in the publication. Y-axis, percentage of HS2/HS3 TPM in MII (GV HS2/HS3 TPM value is set to 100% in each GV–MII pair from the same donor). The absolute clusters’ TPM values are given in Supplementary Table 2.

*ACTB* (β-actin) transcript quantity is relatively stable at the end of GV–MII in mouse oocytes^41^. In our experiments (Supplementary Fig. S2), the level of β-actin in cumulus cells and GV oocytes was also relatively comparable. According to *ACTB* quantification of analyzed transcriptomes , the data published by different authors could not be combined. The *ACTB* TPM varied greatly between publications (Supplementary Table 1) due to differences in RNA sample preparation and details of RNA-sequencing protocols. However, within SRR data from one publication a level of β-actin is more stable especially when pairwise comparison (GV vs MII from the same donor) was performed. To compare HS2/HS3 expression levels in GV and MII stages, we used the raw data published by Reyes et al.^22^. The authors published the data on GV and MII oocytes transcriptomes obtained from ten donors. For each donor, GV and MII transcriptomes were accessible. We have performed computational analysis of the data to quantify HS2/HS3 transcription (Fig. 4 and Supplementary Table 2). In GV cells, the HS TPM value was relatively low compared to β-actin (Supplementary Table 1). In 7 out of 10 donors, the quantity of HS2/HS3 transcripts increased in MII as compared to GV oocytes (Fig. 4).

**Figure 5:**
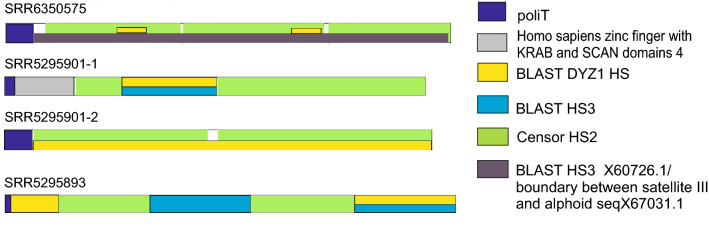
Sequence analysis of all four transcripts assembled from the specified SRR data of published human MI/MII oocyte transcriptomes (see Table 1). The transcripts were analyzed with BLAST and Censor software (a repeat-masking program). The sequences shown in the legend share a high degree of homology with the analyzed sequences (E-value—0–10^−23^).

All the transcripts belonged to the TR DNA families of big satellites. The cDNAs of the most abundant transcripts (Table 1) had signatures typical of HS sequences (Fig. 5), being made of two types of repeats, a pentanucleotide (ATTCC) typical both for HS2 and HS3, and a decanucleotide typical for HS3 (CAACCCGAGT)^2,42^. Only one of them contained a short fragment of a coding sequence (*Homo sapiens* zinc finger with KRAB and SCAN domains 4) at the cDNA 5′ end immediately after the polyT tail. Multiple alignment of all four transcripts and pairwise alignment of sequences revealed that they are not identical and belong to different families of classical satellites (Supplementary, Fig. S3). All the assembled transcripts shared homology (identity > 75%) with different satellite RNA (termed as ‘satellite mRNA’ by the authors) clones obtained by Valgardsdottir et al. (2005)^42^ from heat-shocked HeLa cells (an example is given in Supplementary, Fig S3).

**Figure 6:**
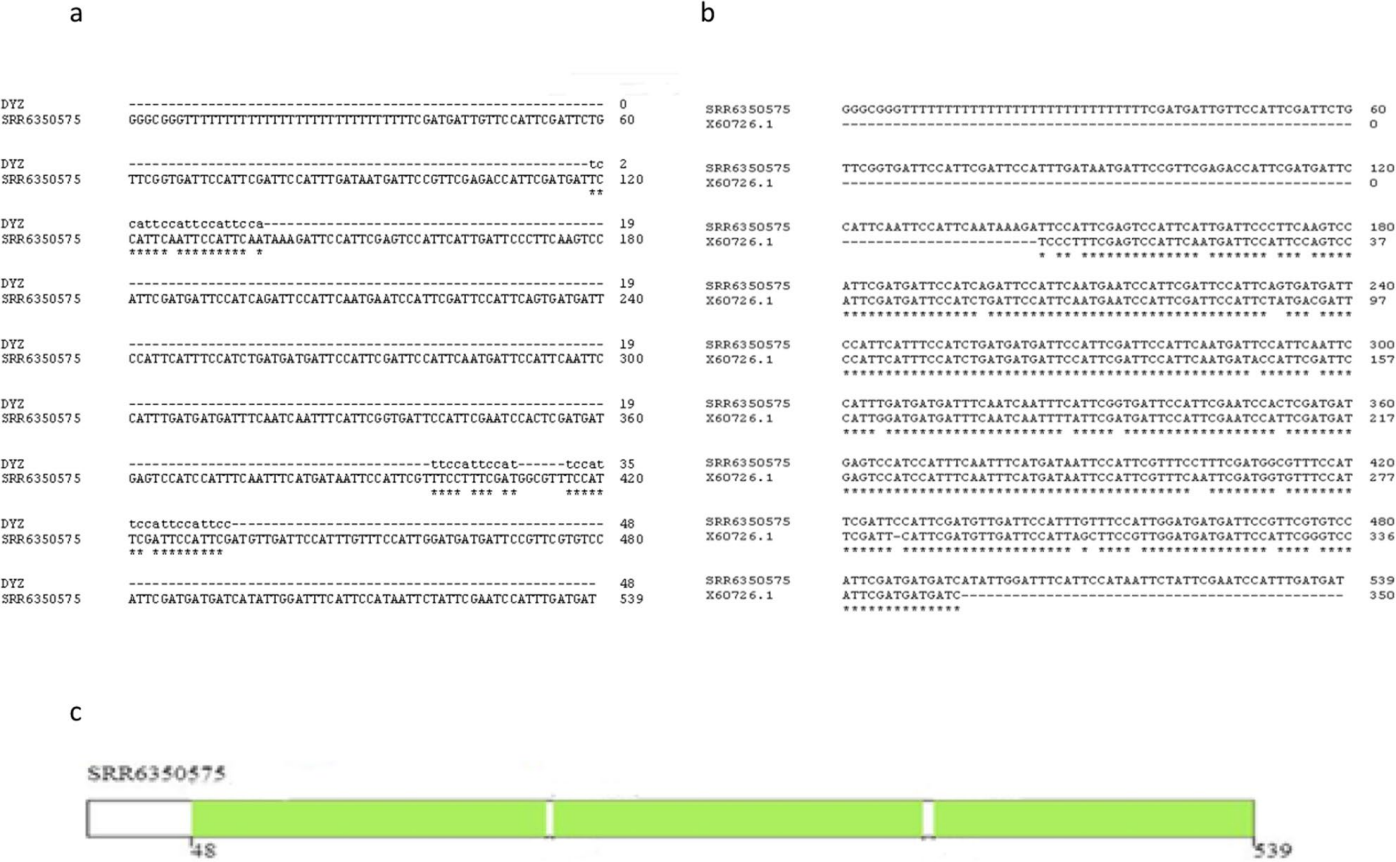
Computational analysis of the transcripts. (**a**) Alignment of the sequence of the oligonucleotide DYZ1 and the sequence of the transcript found in the human transcriptome published by Zhang et al. (2018)^23^ (Accession No. of dataset SRR6350575, see Table 1). (**b**) Alignment of the transcript sequence from the dataset SRR6350575 and the transcript sequence previously detected in tissues of a developing human embryo (Accession No. X60726.1) using another set of primers^44^. (**c**) RepBase analysis of the transcript from the dataset SRR6350575. The transcript contains three parts recognized as HS2/HS3 (green rectangles).

One of the transcripts we assembled from the raw data reads published by Zhang et al. (2018) contained several sites of homology with the DYZ1 probe (Fig. 6a; Table 1). It consisted of periCEN HS2/HS3 blocks. The transcript shared a high degree of homology with the boundary region between CEN alphoid and periCEN HS (Fig. 5). Comparison with RepBase showed that it contained three large HS2/HS3 blocks of approximately equal length (Fig. 6c). It was the only transcript among those shown in Table 1 that contained the GATGAT motif, one of the hallmarks of HS3 of the 1qh region^43^. Moreover, the transcript contained the complete HS3 sequence that we had previously found transcribed in some cancer cell lines, senescent lung fibroblasts and some tissues of a developing human embryo, using different probes and sets of primers^12,44^ (Fig. 6b).

Thus, in late preovulatory oocytes, different HS families of TR DNA are transcribed. The transcript quantity is higher in GV oocytes than in cumulus cells. The HS2/HS3 transcripts content increased further at the end of oogenesis (from GV to MII stages). Both ATTCC and GGAAT strands are transcribed in maturing oocytes. The transcripts are clustered in some membraneless RNP structures that contain helicases DDX4 and DDX5, and are closely associated with mitochondria, being therefore related to Balbiany bodies described previously in early mouse and human oocytes. Further inverstigations are necessary to address the question, whether the appearance of these structures is dependent on the oocyte fate.

## Discussion

### Transcription of HS

In our study, transcripts of a pericentromeric satellites, HS2/HS3, were detected late in human oogenesis, specifically, when an oocyte was leaving the GV stage and entering the MI stage (Fig. 1). The global repression of transcriptional activity occurs just before the resumption of meiosis, at the end of the GV stage when the chromatin begins to condense around the nucleolus^28,29^. It was believed that, during this period, a change in metabolic activity occurred. Transcriptionally quiescent oocytes rely on stored maternal transcripts to sustain the completion of meiosis, fertilization, and early embryonic cleavage stages^30,45,46^.

In our study, both ATTCC and GGAAT strands were transcribed in maturing oocytes. The GGAAT strand transcription was probably delayed according to our data. For computational analysis of transcriptomes, we used publicly available datasets from three different papers^21–23^. All of them, however, provide unstranded RNA-Seq reads. In comparison to strand-specific data, in which a strand of a RNA molecule is preserved, it is not possible to understand whether a read represents the sequence of the molecule itself, or its reverse-complement copy. Thus, de-novo assembly provides contigs with arbitrary strands, and HS2/3 transcripts can be represented as sequences either with “ATTCC” or “GGAAT” pattern. In mouse embryogenesis, transcription of pericentromeric satellites is strand-specific and the switch between chains is regulated in a time-dependent manner^7^. In human, strand-specificity of pericentromeric satellites was demonstrated: in heat shock, the GGAAT chain is predominantly expressed, while in senescent fibroblasts and some cancer lines, the transcribed chain is ATTCC^12,16^.

In FISH experiments, we used probes that revealed signals from both polyadenylated and non-polyadenylated HS2/HS3 RNAs. However, in computational analysis, we analyzed only the quantity of polyadenylated transcripts to avoid contamination with the genome DNA (see “Materials and methods”). Pericentromeric DNA transcripts are known to be polyadenylated in human and mouse^12,42^ though the existence of non-polyadenylated satellite RNA was demonstrated in a rat ascites hepatoma cell line^47^.

We have performed HS2/HS3 quantification by two methods. We confirmed by real-time PCR that the number of transcripts in GV oocytes is higher than in somatic cumulus cells. In the next step, we demonstrated by computational analysis the increase in transcript quantity during the final steps of oocyte maturation. However, it should be taken into account that sequencing of big satellites is complicated due to high GC content. The key problem estimating expression is that pre-centromeric regions containing HS2/HS3 remain unassembled and standard reference-based quantification approaches are not applicable. Although at the next stage of sequence analysis, the remaining fragments of TR are present in SRR data files, these reads are not included in assemblies because they may lead to assembly errors^48^. Nevertheless, the DNA–RNA FISH signal was intense in our experiments (Fig. 1). This fact leads us to assume that the real quantity of HS transcripts in an oocyte is higher than could be expected according to the number of reads in SRR datasets. In other animals, transcription of TR DNA in oogenesis was observed^8,11,49^. Transcription of pericentromere TR DNA in ovaries of *Drosophila*^50^ has been shown. Here we report the existence of HS2/HS3 TR transcripts in human preovulatory GV–MI oocytes for the first time.

### Membraneless mitochondria-associated granules in late maturing oocytes

In mammal embryogenesis, the accumulation of pericentromeric classical satellite transcripts was observed up to the 2–4 cells stage in mice^7,19^ and up to blastocyst stage in human^18^. In mice, downregulation of classical satellite transcription leads to the disruption of the heterochromatinization process, and stops the development of the embryo due to disruption of chromocenters^7^.

In somatic cells, formation of heterochromatin aggregates is necessary to sequestrate some coding regions and proteins^51,52^. Pericentromeric TR transcripts are involved in the formation of RNA–DNA hybrid structures in mice. The authors suggest that it serves to ensure the interaction of pericentromeric chromatin and RNA–protein scaffolds during heterochromatinization^53^. In all these studies describing the HS2/HS3 transcription in somatic cells, transcripts were localized near the pericentromeric sections of the interphase chromosomes. In our study, carried out on preovulatory oocytes, large clusters of HS3 transcripts in MI but not in GV oocytes were detected in the ooplasm far from the chromosomes (Fig. 1e,h,i). This observation suggests that either the transcripts of the pericentromeric satellite perform another function in the oocyte, or the detected clusters play the role of a depot.

The HS2/HS3 transcripts showed a high degree of homology with HS RNAs described in heat-shocked cells, and were termed ‘satellite mRNAs’ by the authors^42^. These transcripts are necessary for the assembly of nuclear stress bodies—membraneless RNP structures associated with pericentromeric heterochromatin during cellular response to stress stimuli, e.g., heat shock^13,16,54^. It was hypothesized that activation of HS3 arrays probably participates in global stress-induced, genome-wide down-regulation of genome expression through transient sequestration of transcription factors^55^. Another set of studies suggests that in cancer cells, HS2/HS3 RNA accumulates in large nuclear foci that are involved in sequestration of different proteins, e.g., MeCP2, Polycomb Group proteins and some master regulators^15,56^. The authors suggested HS2 DNA and RNA have an exceptional capacity to act as molecular sponges and sequester chromatin regulatory proteins into abnormal nuclear bodies in cancer.

Thus, summarizing the data from mouse embryogenesis, heat-shock stressed and cancer cells, it might be assumed that HS transcripts are often involved in the formation of membraneless granules, bodies, etc., with different functions often initiating the structures’ assembly. These RNP structures are used for sequestration and inactivation of different proteins and transcripts. In our experiments, the granules of the transcripts in MI colocalized with DDX5 RNA helicase. The staining pattern of DDX5 overlapped with DDX4 in ooplasm, but not in the nucleus. To date, the presence of only one type of membraneless body, nuage, has been shown in oocytes. Originally nuage was described as a basophilic area of the ooplasm that was necessary to form the animal–vegetal axis in the oocyte of many animals, e.g., *Xenopus* and zebrafish. Later nuage was revealed in the germ cells of many animals, including human and mouse oogonia, and mouse neonatal early oocytes^57–59^. A required attribute of nuage is the presence of RNA helicase, including DDX4 helicase, and various other proteins involved in RNP remodeling and splicing^60–62^. DDX4 is considered to be a marker of these structures because the protein contains intrinsically disordered domain—a domain that is crucial for liquid–liquid phase separation and membraneless structure formation^63^. In rodents, nuage contains molecules that are necessary (1) for the inactivation of retroposons, and (2) for the sequestration of important maternal RNAs, separating them from the ribosomes until their translation is necessary^64^. A peculiar structure, the Balbiani body was described by^33^: it is not surrounded by a membrane and usually consists of an aggregate of mitochondria, often interspersed with the electron-dense nuage, endoplasmic reticulum cisternae, and Golgi complexes^33^. The Balbiani body was observed in oocytes of all vertebrates including human and mouse^33,57^. In *Xenopus* and zebrafish, the Balbiani bodies are necessary to mark the vegetal pole of an egg. In mouse and humans, it is not known whether these structures are involved in polarity formation. Balbiani bodies of mammals share many features with stress bodies of other cell types. Stress bodies are distinct cellular granules containing mRNAs and enzymes that mediate mRNA turnover or storage. Therefore, it was hypothesized that the role of the Balbiani body might be to protect RNAs from being degraded and/or to prevent expression or activation of patterning molecules before they are needed. This structure also provides a mechanism to spatially restrict their activity. Notably, the maternal mRNAs required at later stages to establish the germline of the embryo are localized to the Balbiani body^65^. However, in mammals, the Balbiani bodies were first observed in primordial follicles (e.g., by Young et al., 1999^66^). Later, they were demonstrated in mouse germ cell cysts and primordial follicles^67^. The mouse Balbiani body forms in germ cell cysts just before primordial follicle formation (around the birth time, when oocytes are in late pachytene-early diplotene stages) and persists briefly in young primordial follicles. In growing follicles, mitochondria and ER disperse, and a well-defined Balbiani body is no longer found^57,68^. However, the the polar cortical area of basophilic ooplasm persists at least until the completion of MI^66^. In mice, some nuage proteins form transient, RNA-containing aggregates in the fully-grown SN oocytes. These aggregates, referred to as mouse oocyte P-bodies, disperse during oocyte maturation, consistent with recruitment of maternal mRNAs that occurs during this time^69^. The nuage marker protein MVH (DDX4) is detectable in mouse oocytes of adult animals from the primordial follicle stage until the beginning of the formation of antral spaces in the follicular stage^70^. In our study carried out on human samples, the protein was present in maturing MI oocytes taken from antral follicles. Thus, the timing of DDX4-positive membraneless aggregates assembly is different in human and mouse oogenesis. However, in mouse, the existence of distinct clusters of polyA transcripts was established in GV–MII^71^. Some authors reported, upon the disappearance of germ cell granules or P-bodies in mammalian oocytes, a new mRNA storage region appears close to the SN oocyte cortex^24^.

To date, no membraneless granules associated with mitochondria have been described in oocytes at the late stages of maturation. However, some of the mRNA, non-coding RNA and proteins still need to be sequestrated. Mitochondria-associated membraneless coacervate bodies assembled on the base of pericentromeric transcripts might be a place for such spatial sequestration.

## Materials and methods

### Phytohemagglutinin-stimulated lymphocytes

Peripheral blood samples (500 μl) of healthy donors were transferred to centrifuge tubes (15 ml) with 4.5 ml of RPMI medium containing 10% fetal bovine serum (FBS) and a mixture of penicillin (50 U) and streptomycin (50 μg). Phytohemagglutinin (PHA) was added at a concentration of 10 µg/ml. The tubes were incubated at 37 °C in an atmosphere of 5% CO_2_ for 72 h. Then, colchicine (5 μg/ml) was added to the cells. After an hour, the samples were centrifuged (200 × g, 10 min) and the precipitate was resuspended in 10 ml of 0.075 M KCl, heated to 37 °C, and incubated for 15 min. The cells were centrifuged; the pellet was resuspended in the ethanol/glacial acetic acid fixative, centrifuged, again resuspended in 500 μl of the same fixative, and spread onto slides. The slides were air-dried and stored at room temperature.

### Preovulatory human oocytes

**Figure 7:**
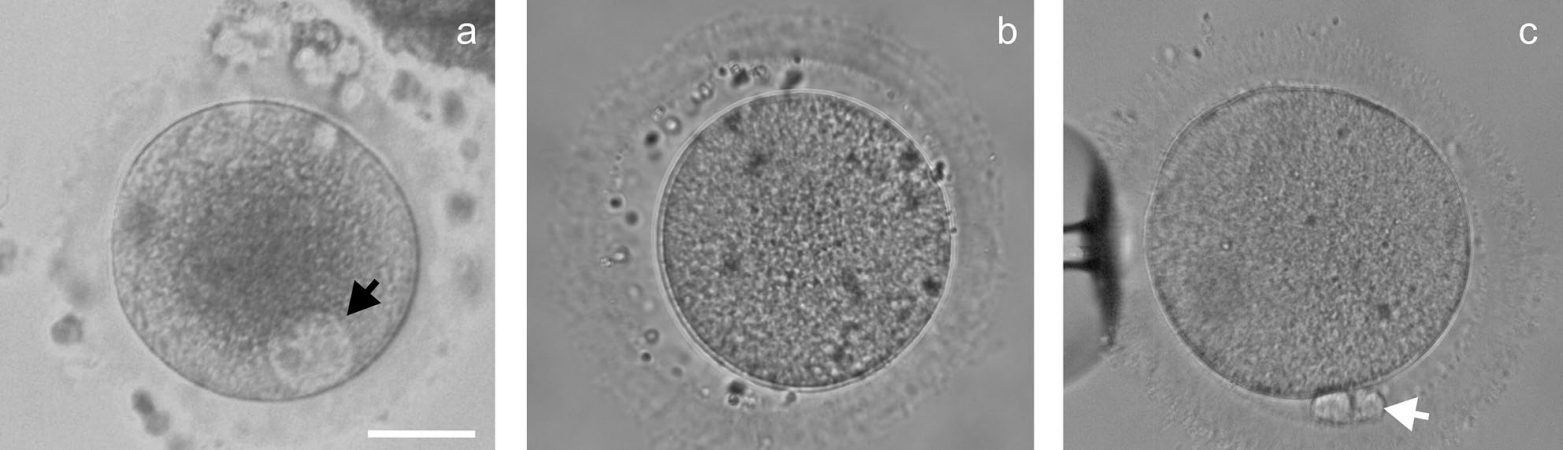
Oocytes after transvaginal sonographically controlled follicle punctures and cumulus removal. The images were taken during initial examination after punctures. GV (**a**) and MI (**b**) oocytes are excluded from the egg donation program. Oocytes at MII stage (**c**) are used in assisted reproductive technologies. Black arrow—the nucleus with a kariosphere, white arrow—a polar body. Scale bar: 50 μm.

Oocytes (GV, MI, total n = 68) were obtained from healthy donors, who were included in the oocytes donation program and treated according to standard ovarian stimulation protocols. Antral follicles were collected with a needle during transvaginal, sonographically controlled, follicle punctures. The aspirates contained GV, MI and MII oocytes. The quality of hyaluronidase-treated cumulus-free oocytes was evaluated via stereomicroscopy investigation. The oocytes were sorted into suitable and unsuitable classes for the donation program. Oocytes were considered mature and suitable for donation if they reached the MII stage (with a prominent polar body and an absent nucleus), had a diameter of 125–135 μm and met morphological criteria according to the Ava-Peter-Scandinavia clinics’ Standard Operational Protocols (Fig. 7).

GV and MI oocytes without morphological abnormalities were excluded from the egg donation program and were used in the present study if an informed consent was signed by the donor. The degree of meiotic cell maturity was evaluated according to the following classification: in the absence of a polar body the oocyte was judged to be at the GV stage if the nucleus was visible in the cell cytoplasm, and at the MI stage if the nucleus was not seen (Fig. 7). During immunocytochemical staining and FISH experiments, we could distinguish GVBD and MI oocytes after staining oocytes with DAPI. If a nuclear membrane was not revealed, but a karyosphere was still visible, the oocyte was considered as GVBD. An oocyte with a clearly visible metaphase plate was identified as an MI oocyte.

After debris removal and microscopic examination, GV and MI oocytes were placed in 1 μl of manipulation medium (CooperSurgical, USA) on a glass slide and fixed with 40 μl of a cold ethanol/glacial acetic acid fixative. The slides were air dried and stored at room temperature. At least five oocytes were used for each experiment.

### DNA probe

The Cy3-labelled oligonucleotide probe DYZ1: 5′-TCCATTCCATTCCATTCCATTCCATTCCATTCCATTCCATTCCATTCC-3′ was used for DNA–DNA FISH and DNA–RNA FISH. The sequence was originally described as being located in an array of pericentromere and long arm TR DNA on the Y chromosome (https://eul1db.unice.fr//Variant.jsp?showVariant=srip142266) (Supplementary Fig. S4a)^72^. However, the sequence is present in the pericentromeric regions of most chromosomes in the arrays of HS2 and HS3 (Supplementary Fig. S4b).

The DYZ1 probe is based on ATTCC repeat. HS2/HS3 can be transcribed from both chains. Under heat shock, the GGAAT chain is predominantly expressed, while in senescent fibroblasts, the transcribed chain is ATTCC^12,16^. A FAM-conjugated probe, PCT14, containing a sequence from a chromosome 14 subfamily of HS3 DNA (pTRS-63), was designed (5′-TCAACCCGAGTGTGTTTCATTGGAATGGAACGAAAGGAAGGGAATGGGGT-3′) that was based on GGAAT repeat and its variations. Despite the subfamily being chromosome-specific, the probe hybridized with pericentromeric regions of most chromosomes (Supplementary Fig. S1). RNA–FISH with two probes (DYZ1, red, and PCT14, green) was carried out in order to study, whether the HS2/3 transcription is strand-specific.

### DNA–DNA and DNA–RNA FISH

The distribution of DYZ1 and PCT14 hybridization sites in human preovulatory oocytes at the GV and MI stages, as well as on metaphase spreads of human lymphocytes, was studied with fluorescence in-situ hybridization (FISH).

Fixed preparations (lymphocyte spreads or oocytes) were placed in 2 × sodium saline citrate (SSC) for 30 min at 37 °C and were then dehydrated in an alcohol series and air-dried. The denaturation step was performed in 30% formamide for DYZ1, in 70% for PCT14 in 2 × SSC, pH = 7.0 for 7 min at 78 °C. The oocytes were then dehydrated again. For DNA–RNA FISH, the denaturation step was omitted. The hybridization mixture (1 µg/ml Cy3-DYZ1 or FAM-PCT14, 30% formamide for DYZ1 or 50% for PCT14, 2 × SSC, 10% dextransulfate, 5 × Denhardt's solution, 0.5 mg/ml salmon sperm DNA) was preheated to 41 °C and applied to the slides. The hybridization was performed at 41 °C (DYZ1) or 37 °C (PCT14) in a humid chamber for 5 h. The slides were then washed in 2 × SSC for 10 min at 41 °C, in 1 × SSC for 10 min at room temperature, in 0.5 × SSC and 0.25 × SSC for 5 min each at room temperature. The slides were then rinsed with distilled water and mounted in a medium containing an antifade agent and DAPI.

As a negative control, some of the oocytes were pretreated with RNase A before the dehydration step. The slides were incubated in a 200 μg/ml RNase A in 2 × SSC, pH = 7.0 for 1 h at 37 °C, and then washed in 2 × SSC for 10 min.

### Immuno-FISH and double immunostaining

To evaluate the distribution of human satellite transcripts with respect to helicase DDX5, a DNA–RNA FISH was combined with indirect immunocytochemical staining (immuno–DNA–RNA FISH).

After DNA–RNA FISH described above, oocyte preparations were kept in 5% bovine serum albumin in 1 × phosphate buffered saline (PBS) for 1 h. A polyclonal goat anti-DDX5 antibody (AB) (1:150, Abcam, #ab10261) was applied. The slides were then washed in PBS containing 0.02% Tween-20 (PBST) three times, 10 min each, and a correspondent secondary AB conjugated with Alexa 488 was applied for 1 h. After this, the preparations were again washed, rinsed with water and mounted in an antifade medium with DAPI.

The spatial distribution of DDX5 with respect to mitochondria was studied in double immunostaining experiments. The oocytes were fixed in a drop of 4% paraformaldehyde in PBS then treated with Triton X-100 (0.05% for 30 min) and washed in PBS. Fixed and permeabilized oocytes were transferred into a drop of PBS containing 5% bovine serum albumin. All the subsequent procedures were also carried out in drops, in Petri dishes placed in a humidified chamber. AntiDDX5 staining was performed with the AB against DDX5 conjugated with Alexa488 (1:200, Abcam, #ab199226). When this step was completed, the cells were washed and mitochondria were visualized with the AB against import receptors Tom20 of mitochondrial preprotein translocases of the outer membrane (Tom). The primary AB (Santa-Cruz, #sc-17764) was applied at 1:100 for 1 h. The oocytes were then washed in PBS containing 0.02% Tween-20 (PBST) three times, 10 min each, and a correspondent secondary AB conjugated with Cy3 was applied for 1 h. After this, the preparations were again washed, rinsed with water, put onto a slide, and mounted in an antifade medium with DAPI.

The colocalization of DDX5 and DDX4 was also studied in double immunostaining experiments. After immuno-DNA–RNA FISH described above, oocyte preparations were kept in 5% bovine serum albumin in 1 × phosphate buffered saline (PBS) for 1 h. AntiDDX4 staining was performed with the mouse monoclonal AB against DDX4 conjugated with Alexa 647 (1:200, Abcam, # ab196708). The slides were then washed in PBST three times, 10 min each. After this, the preparations were rinsed with water and mounted in an antifade medium with DAPI.

### Confocal microscopy

The images were acquired with an Olympus FV3000 confocal microscope (Nikon, Japan). To detect DAPI, FITC (or Alexa488), Cy3 and Alexa 647, the 405, 488, 561 and 640 nm diode lasers were used for excitation, respectively. The cells were sectioned along the z-axis at a 0.8 µm interval. Three-dimensional reconstruction, based on the series of optical sections, was performed using the built-in functions of the Olympus FV3000 microscope software. The same software was used for further image processing.

### RNA isolation and real-time PCR analysis

GV oocytes were obtained as described above in the *Preovulatory human oocytes* section and vitrified using the Cryotop open system (Kitazato, Japan). Cumulus cells (from five donors) were also collected, transferred to a plastic cryotube, and frozen in liquid nitrogen vapor until the day of experiment. On the day of experiment, 60 oocytes (from seven donors) were thawed according to the Cryotop thawing protocol. Total RNA samples from oocytes and cumulus cells were isolated using a GenElute Mammalian Total RNA Miniprep Kit. The RNA concentration was measured with a spectrophotometer (NanoQuant Infinite F200 PRO, TECAN). Total RNA was treated by Dnase I. PoliT Complementary DNA (cDNA) was synthesized from total purified RNA using M-MuLV–RH reverse transcription kit.

qPCR reaction was performed using CFX96 Real-Time System (Bio-Rad Laboratories, USA) with 100 ng of cDNA quantified with Maxima SYBR Green/ROX qPCR Master Mix (2X). The HS2/HS3 sequences were amplified using the following primers: 5′-CGTTTCCTTTCGATGGCGTT-3′ as a forward and 5′-TGAAATCCAATATGATCATCATCGAA-3′ as a reverse primer. The primer set was designed using a transcript from a transcriptome published by Zhang et al. (2018) (Table 1). The qPCR conditions were: an initial denaturation step at 95 °C for 3 min; 40 cycles of 10 s at 95 °C and 30 s at 58 °C. Melting curve analyses were performed at the end of each PCR assay to verify the specificity of the PCR products. mRNA expression levels were calculated by the 2^−ΔΔCt^ method with the levels of gene expression normalized to the two genes: GAPdH (Forward: 5′-AGGTCGGAGTCAACGGATTT-3′; Reverse: 5′-TTCCCGTTCTCAGCCTTGAC-3′; designed by the authors) and β-actin (Forward: 5′-ATTGCCGACAGGATGCAGA-3′; Reverse: 5′-GAGTACTTGCGCTCAGGAGGA-3′; designed by Baldion et al.^73^).

### Bioinformatic analysis of the transcriptome of preovulatory oocytes

Recently, several studies on transcriptomes of the human preovulatory oocyte were reported^21–23^. We analyzed several publicly available RNA-Seq datasets of healthy donors from these studies (accession numbers are given in Table 1 and Supplementary Table 1) in order to check the presence of sequences containing homology regions with the DYZ1 probe. Raw reads were polyA-trimmed using Cutadap^74^. For additional quality control, reads were mapped to the Human GRCh38 reference genome with STAR aligner^75^ and checked for DNA contamination using Qualimap2^76^. Overall percentage of intergenic reads varied within the typical 5.5–8.5% range, which suggests no substantial DNA contamination was present in the analyzed datasets. Trimmed reads were further assembled using rnaSPAdes^77^ and the assembled transcripts were screened for the presence of the DYZ1 sequence using the special mode of the BLAST aligner^78^ designed for short sequences (-task blastn-short option). For further analysis, only transcripts containing highly confident matches were selected (exact match with half of the DYZ1 sequence). Since both trimming and assembly software remove poly-A/T tails, initial untrimmed reads were mapped back to these transcripts using minimap2^39^ to check for the presence of poly-A tails. As a result, polyadenylated transcripts from different datasets were detected with length ranging from 300 to 640 bp. These transcripts were clustered using UCLUST into seven clusters. A representative consensus sequence was computed for each cluster and further quantified using Kallisto^40^. In addition, we estimated the total expression for three known reference HS2/3 sequences—X60726.1, S90110.1, X82942.1 to check whether the quantity trends for clusters consensuses corresponded to trends for already-known sequences.

BLAST, CLUSTAL-O, Censor and EMBOSS Needle software were used for the transcripts analysis: finding regions of similarity, multiple sequence alignment, repeats masking and pairwise sequence comparison, respectively^78–81^.

*ACTB* (β-actin) transcripts quantity is relatively stable at the end of GV-MII in human (our own data, see the Results section) and mouse^41^ oocytes. *HBA1* (hemoglobin subunit alpha 1) is not expressed in oocytes. Therefore ACTB (mRNA NCBI Reference Sequence: NCBI Reference Sequence: NM_001101.5) and HBA1 (mRNA NCBI Reference Sequence: NM_000558.5) were used as positive and negative controls for quantification.

According to *ACTB* quantification (Supplementary Table 1), data from transcriptomes published by different authors cannot be combined for quantity comparison between oocytes at different stages. To compare HS2/HS3 expression levels in GV and MII stages, we performed the same analysis as described above for all 20 available samples published by Reyes et al.^22^. In this publication, transcriptomes of GV and MII oocytes from the same donors are given. Therefore, for the donors, the pairwise comparison of HS2/HS3 transcript quantity in GV and MII oocytes can be performed.

### Statistical analysis

Real-time PCR reactions were performed in triplicate. The experiment was repeated three separate times. Statistical analysis was performed using GraphPad Prism7. Data are presented as mean ± standard deviation (SD). For comparison of two groups, normally distributed data were analysed by t-test. Statistical significance was denoted by **p-value < 0.01.

### Ethical approval

All the oocytes were obtained from donors according to the order of the Russian Federation Ministry of Health of August 11, 2017, no. 517n, and in line with the Word Medical Association Helsinki Declaration (Declaration of Helsinki: Ethical Principles for Medical Research Involving Human Subjects, including amendments made by the 64th Meeting of the WMA in Fortaleza, Brazil, October 2013). The local ethical committee of Ava-Peter-Scandinavia clinics approved the study (#11/22-12-2016). Written informed consent was obtained from each donor enrolled in the study.

## Supplementary information

Supplementary Information.

## References

[1] Richard G-F, Kerrest A, Dujon B (2008). Comparative genomics and molecular dynamics of DNA repeats in eukaryotes. Microbiol. Mol. Biol. Rev..

[2] Prosser J, Frommer M, Paul C, Vincent PC (1986). Sequence relationships of three human satellite DNAs. J. Mol. Biol..

[3] Therkelsen AJ, Nielsen A, Kølvraa S (1997). Localisation of the classical DNA satellites on human chromosomes as determined by primed in situ labelling (PRINS). Hum. Genet..

[4] Jeanpierre M (1994). Human satellites 2 and 3. Ann. Genet..

[5] Tagarro I, Fernández-Peralta AM, González-Aguilera JJ (1994). Chromosomal localization of human satellites 2 and 3 by a FISH method using oligonucleotides as probes. Hum. Genet..

[6] Li H (2019). Identifying centromeric satellites with dna-brnn. Bioinformatics.

[7] Probst AV (2010). A strand-specific burst in transcription of pericentric satellites is required for chromocenter formation and early mouse development. Dev. Cell.

[8] Gaginskaya E, Kulikova T, Krasikova A (2009). Avian lampbrush chromosomes: a powerful tool for exploration of genome expression. Cytogenet. Genome Res..

[9] Kuznetsova IS (2014). Primary analysis of repeat elements of the Asian seabass (*Lates calcarifer*) transcriptome and genome. Front. Genet..

[10] Solovei IV, Joffe BI, Gaginskaya ER, Macgregor HC (1996). Transcription of lampbrush chromosomes of a centromerically localized highly repeated DNA in pigeon (Columba) relates to sequence arrangement. Chromosome Res..

[11] Trofimova I, Krasikova A (2016). Transcription of highly repetitive tandemly organized DNA in amphibians and birds: a historical overview and modern concepts. RNA Biol..

[12] Enukashvily NI, Donev R, Waisertreiger IS-R, Podgornaya OI (2007). Human chromosome 1 satellite 3 DNA is decondensed, demethylated and transcribed in senescent cells and in A431 epithelial carcinoma cells. Cytogenet. Genome Res..

[13] Rizzi N (2004). Transcriptional activation of a constitutive heterochromatic domain of the human genome in response to heat shock. Mol. Biol. Cell.

[14] Enukashvily NI, Malashicheva AB, Waisertreiger IS-R (2009). Satellite DNA spatial localization and transcriptional activity in mouse embryonic E-14 and IOUD2 stem cells. Cytogenet. Genome Res..

[15] Hall LL (2017). Demethylated HSATII DNA and HSATII RNA foci sequester PRC1 and MeCP2 into cancer-specific nuclear bodies. Cell Rep..

[16] Jolly C (2004). Stress-induced transcription of satellite III repeats. J. Cell Biol..

[17] Saksouk N, Simboeck E, Déjardin J (2015). Constitutive heterochromatin formation and transcription in mammals. Epigenetics Chromatin.

[18] Yandım C, Karakülah G (2019). Expression dynamics of repetitive DNA in early human embryonic development. BMC Genom..

[19] Santenard A (2010). Heterochromatin formation in the mouse embryo requires critical residues of the histone variant H3.3. Nat. Cell Biol..

[20] Veselovska L (2015). Deep sequencing and de novo assembly of the mouse oocyte transcriptome define the contribution of transcription to the DNA methylation landscape. Genome Biol..

[21] Ferrero H (2019). Single-cell RNA sequencing of oocytes from ovarian endometriosis patients reveals a differential transcriptomic profile associated with lower quality. Hum. Reprod..

[22] Reyes JM (2017). Differing molecular response of young and advanced maternal age human oocytes to IVM. Hum. Reprod..

[23] Zhang Y (2018). Transcriptome landscape of human folliculogenesis reveals oocyte and granulosa cell interactions. Mol. Cell.

[24] Christou-Kent M, Dhellemmes M, Lambert E, Ray PF, Arnoult C (2020). Diversity of RNA-binding proteins modulating post-transcriptional regulation of protein expression in the maturing mammalian oocyte. Cells.

[25] Szöllösi MS, Debey P, Szöllösi D, Rime H, Vautier D (1991). Chromatin behaviour under influence of puromycin and 6-DMAP at different stages of mouse oocyte maturation. Chromosoma.

[26] Debey P (1993). Competent mouse oocytes isolated from antral follicles exhibit different chromatin organization and follow different maturation dynamics. Mol. Reprod. Dev..

[27] Bogolyubov DS (2018). Karyosphere (Karyosome): a peculiar structure of the oocyte nucleus. Int. Rev. Cell Mol. Biol..

[28] De La Fuente R, Eppig JJ (2001). Transcriptional activity of the mouse oocyte genome: companion granulosa cells modulate transcription and chromatin remodeling. Dev. Biol..

[29] Shishova KV, Khodarovich YM, Lavrentyeva EA, Zatsepina OV (2015). High-resolution microscopy of active ribosomal genes and key members of the rRNA processing machinery inside nucleolus-like bodies of fully-grown mouse oocytes. Exp. Cell Res..

[30] Virant-Klun I, Knez K, Tomazevic T, Skutella T (2013). Gene expression profiling of human oocytes developed and matured in vivo or in vitro. Biomed. Res. Int..

[31] Anderson P, Kedersha N (2006). RNA granules. J. Cell Biol..

[32] Meikar O (2014). An atlas of chromatoid body components. RNA.

[33] Kloc M, Bilinski S, Etkin LD (2004). The Balbiani body and germ cell determinants: 150 years later. Curr. Top. Dev. Biol..

[34] Castrillon DH, Quade BJ, Wang TY, Quigley C, Crum CP (2000). The human VASA gene is specifically expressed in the germ cell lineage. Proc. Natl. Acad. Sci. USA.

[35] Enukashvily N, Donev R, Sheer D, Podgornaya O (2005). Satellite DNA binding and cellular localisation of RNA helicase P68. J. Cell Sci..

[36] Tanious FA, Veal JM, Buczak H, Ratmeyer LS, Wilson WD (1992). DAPI (4’,6-diamidino-2-phenylindole) binds differently to DNA and RNA: minor-groove binding at AT sites and intercalation at AU sites. Biochemistry.

[37] Biase FH, Fonseca Merighe GK, Santos Biase WKF, Martelli L, Meirelles FV (2008). Global poly(A) mRNA expression profile measured in individual bovine oocytes and cleavage embryos. Zygote.

[38] Caetano LC (2019). Validation of reference genes for gene expression studies in bovine oocytes and cumulus cells derived from in vitro maturation. Anim. Reprod..

[39] Li H (2018). Minimap2: pairwise alignment for nucleotide sequences. Bioinformatics.

[40] Bray NL, Pimentel H, Melsted P, Pachter L (2016). Near-optimal probabilistic RNA-seq quantification. Nat. Biotechnol..

[41] Oliveri RS (2007). Evaluation in mammalian oocytes of gene transcripts linked to epigenetic reprogramming. Reproduction.

[42] Valgardsdottir R (2005). Structural and functional characterization of noncoding repetitive RNAs transcribed in stressed human cells. Mol. Biol. Cell.

[43] Podgornaya O, Dey R, Lobov I, Enukashvili N (2000). Human satellite 3 (HS3) binding protein from the nuclear matrix: isolation and binding properties. Biochim. Biophys. Acta (BBA) Mol. Cell Res..

[44] Kuznetzova T (2012). Localisation and transcription of human chromosome 1 pericentromeric heterochromatin in embryonic and extraembryonic tissues. Med. Genet..

[45] Ma J-Y (2013). Maternal factors required for oocyte developmental competence in mice: transcriptome analysis of non-surrounded nucleolus (NSN) and surrounded nucleolus (SN) oocytes. Cell Cycle.

[46] Tomek W, Torner H, Kanitz W (2002). Comparative analysis of protein synthesis, transcription and cytoplasmic polyadenylation of mRNA during maturation of bovine oocytes in vitro. Reprod. Domest. Anim..

[47] Miyahara M, Sumiyoshi H, Yamamoto M, Endo H (1985). Strand specific transcription of satellite DNA I in rat ascites hepatoma cells. Biochem. Biophys. Res. Commun..

[48] Tørresen OK (2019). Tandem repeats lead to sequence assembly errors and impose multi-level challenges for genome and protein databases. Nucl. Acids Res..

[49] Macgregor HC (1979). In situ hybridization of highly repetitive DNA to chromosomes of *Triturus cristatus*. Chromosoma.

[50] Usakin L (2007). Transcription of the 1.688 satellite DNA family is under the control of RNA interference machinery in Drosophila melanogaster ovaries. Genetics.

[51] Brown KE (1997). Association of transcriptionally silent genes with Ikaros complexes at centromeric heterochromatin. Cell.

[52] Fedorova E, Zink D (2008). Nuclear architecture and gene regulation. Biochim. Biophys. Acta (BBA) Mol. Cell Res..

[53] Velazquez Camacho O (2017). Major satellite repeat RNA stabilize heterochromatin retention of Suv39h enzymes by RNA-nucleosome association and RNA:DNA hybrid formation. Elife.

[54] Metz A, Soret J, Vourc’h C, Tazi J, Jolly C (2004). A key role for stress-induced satellite III transcripts in the relocalization of splicing factors into nuclear stress granules. J. Cell. Sci..

[55] Biamonti G, Vourc’h C (2010). Nuclear stress bodies. Cold Spring Harb. Perspect. Biol..

[56] Brückmann NH, Pedersen CB, Ditzel HJ, Gjerstorff MF (2018). Epigenetic reprogramming of pericentromeric satellite DNA in premalignant and malignant lesions. Mol. Cancer Res..

[57] Kloc M (2008). Mouse early oocytes are transiently polar: three-dimensional and ultrastructural analysis. Exp. Cell Res..

[58] Batalova F, Parfenov V (2003). Immunomorphological localization of Vasa protein and pre-mRNA splicing factors in *Panorpa communis* trophocytes and oocytes. Cell Biol. Int..

[59] Kellokumpu-Lehtinen PL, Söderström KO (1978). Occurrence of nuage in fetal human germ cells. Cell Tissue Res..

[60] Arkov AL, Ramos A (2010). Building RNA-protein granules: insight from the germline. Trends Cell Biol..

[61] Linder P, Stutz F (2001). mRNA export: travelling with DEAD box proteins. Curr. Biol..

[62] Rocak S, Linder P (2004). DEAD-box proteins: the driving forces behind RNA metabolism. Nat. Rev. Mol. Cell Biol..

[63] Hondele M (2019). DEAD-box ATPases are global regulators of phase-separated organelles. Nature.

[64] Lim AK, Knowles BB (2015). Controlling endogenous retroviruses and their chimeric transcripts during natural reprogramming in the oocyte. J. Infect. Dis..

[65] Marlow FL (2010). Maternal Control of Development in Vertebrates: My Mother Made Me Do It!.

[66] Young JK, Allworth AE, Baker JH (1999). Evidence for polar cytoplasm/nuage in rat oocytes. Anat. Embryol..

[67] Pepling ME, Wilhelm JE, O’Hara AL, Gephardt GW, Spradling AC (2007). Mouse oocytes within germ cell cysts and primordial follicles contain a Balbiani body. Proc. Natl. Acad. Sci. USA.

[68] Borum K (1961). Oogenesis in the mouse. A study of the meiotic prophase. Exp. Cell Res..

[69] Flemr M, Ma J, Schultz RM, Svoboda P (2010). P-body loss is concomitant with formation of a messenger RNA storage domain in mouse oocytes. Biol. Reprod..

[70] Toyooka Y (2000). Expression and intracellular localization of mouse Vasa-homologue protein during germ cell development. Mech. Dev..

[71] Trapphoff T (2016). Postovulatory aging affects dynamics of mRNA, expression and localization of maternal effect proteins, spindle integrity and pericentromeric proteins in mouse oocytes. Hum. Reprod..

[72] Lee E (2012). Landscape of somatic retrotransposition in human cancers. Science.

[73] Baldión PA, Velandia-Romero ML, Castellanos JE (2018). Odontoblast-like cells differentiated from dental pulp stem cells retain their phenotype after subcultivation. Int. J. Cell Biol..

[74] Martin M (2011). Cutadapt removes adapter sequences from high-throughput sequencing reads. EMBnet.journal.

[75] Dobin A (2013). STAR: ultrafast universal RNA-seq aligner. Bioinformatics.

[76] Okonechnikov K, Conesa A, García-Alcalde F (2016). Qualimap 2: advanced multi-sample quality control for high-throughput sequencing data. Bioinformatics.

[77] Bushmanova E, Antipov D, Lapidus A, Prjibelski AD (2019). rnaSPAdes: a de novo transcriptome assembler and its application to RNA-Seq data. Gigascience.

[78] Camacho C (2009). BLAST+: architecture and applications. BMC Bioinform..

[79] Kohany O, Gentles AJ, Hankus L, Jurka J (2006). Annotation, submission and screening of repetitive elements in Repbase: RepbaseSubmitter and Censor. BMC Bioinform..

[80] Needleman SB, Wunsch CD (1970). A general method applicable to the search for similarities in the amino acid sequence of two proteins. J. Mol. Biol..

[81] Sievers F (2011). Fast, scalable generation of high-quality protein multiple sequence alignments using Clustal Omega. Mol. Syst. Biol..

