# Supplementary material for: Human pericentromeric tandemly repeated DNA is transcribed at the end of oocyte maturation and is associated with membraneless mitochondria-associated structures
Source: Sci Rep. 2020 Nov 12;10:19634. doi: 10.1038/s41598-020-76628-8 (PMC7665179; doi:10.1038/s41598-020-76628-8)
Supplement: Supplementary file 1 — Supplementary Information. [file 41598_2020_76628_MOESM1_ESM.pdf]

Supplementary information for

Human pericentromeric tandemly repeated DNA is transcribed at the end of oocyte maturation and is associated with membraneless mitochondria-associated structures

Dobrynin M.A.[1], Korchagina N.M.[2,4], Prjibelski A.D.[3], Shafranskaya D.[3], Ostromyshensky D.I.[1] Shunkina K.[2], Stepanova I.[1], Kotova A.V.[1,5], Podgornaya O.I.[1, 4], Enukashvily N.I.[1,5]*

[1.] Institute of Cytology RAS
[2.] Ava-Peter – Scandinavia Assisted Reproductive Technology Clinic
[3.] Center for Algorithmic Biotechnology, St. Petersburg State University
[4] Faculty of Biology, St. Petersburg State University
[5.] North-Western State Medical University named after I.I. Mechnikov

* - n.enukashvily@incras.ru

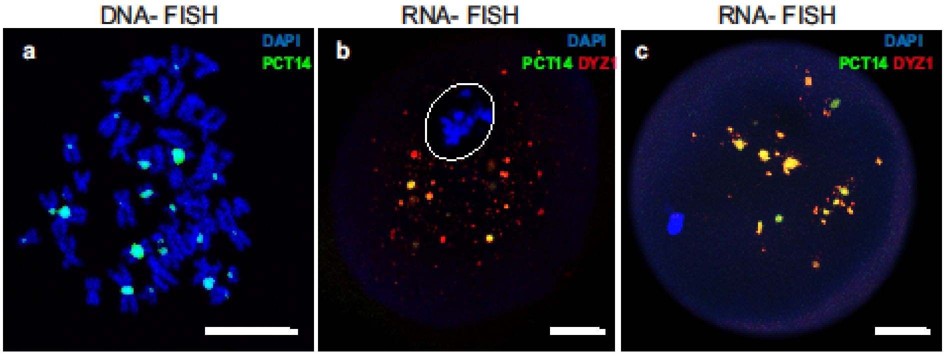

Supplementary Fig. S1. . Spatial distribution of PCT14 DNA (a) and RNA (b-c) relative to DYZ1 RNA (b-c). The DYZ1 probe is basically an "ATTCC" repeat, while PCT14 is based on GGAAT repeat. . (a) Localization of HS2/HS3 DNA probed with PCT14 (green) in metaphase spreads of human lymphocytes. RNA-FISH with two probes (DYZ1, red, and PCT14, green) was carried out in order to study, whether the HS2/3 transcription is strain-specific in human GV (b) and MI (c) oocytes. The nucleus in (b) was encircled with dotted white line after the merge with brightfield data (not included in the panel) Chromatin was counterstained with DAPI (blue). Scale bars: (5 µm) (a) and (20 µm) (b,c).

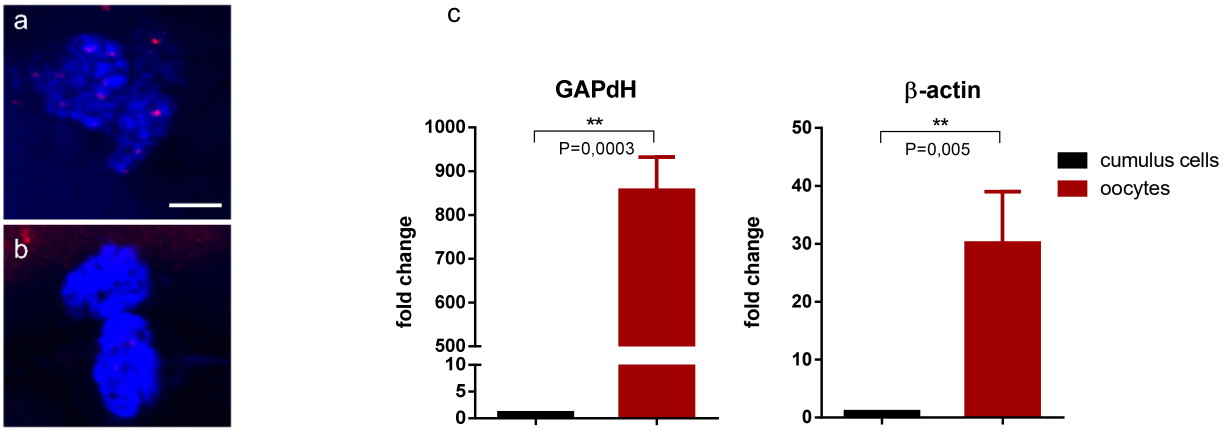

Supplementary Fig. S2. Relative expression of HS2/HS3 in human oocytes and cumulus cells. Cumulus cells express little (a) or no (b) HS2/HS3 transcripts as shown by RNA-FISH with the DYZ1 probe (red). The real-time PCR data were calculated by the $2^{-\Delta\Delta Ct}$ method with the levels of gene expression normalized to the two genes: GAPdH or β-actin (c). Y-axis: fold change (mean ± SD) between cumulus cells (obtained from 5 donors) and oocytes (obtained from 7 donors, total n=60). ** - p<0.01, t-test. Nuclei in (a, b) were counterstained with DAPI. Scale bar: 50 μm.

Supplementary Table 1. Quantification (in Transcripts Per Million, TPM) of HS2/HS3 transcription in published oocyte transcriptomes

Publication	Reyes et al., 2017 (6 of 20 transcriptomes)						Zhang et al., 2018	Ferrero et al., 2019		
Sample ID (SRR No)	5295892	5295900	5295904	5295893	5295901	5295905	6350575	8446791	8446800	8446805
Stage	GV	GV	GV	MII	MII	MII	Preovulatory	MII	MII	MII
Donor age	29	20	42	29	20	42	N/A	24	18	27
Total clusters' TPM	**2,61721**	**16,950211**	**2,440013**	**12,680011**	**84.924001**	**6,582101**	**13,00845**	**4,712133**	**8,850269**	**14,167788**
Total References sum TPM	**0,89045**	**3,03511**	**0,631223**	**5,886452**	**34,79495**	**2,411017**	**12,047245**	**1,080532**	**2,37208**	**3,64405**
ACTB	152,431	243,856	189,985	172,468	364,043	245,628	4416,17	46,9432	33,9912	50,3112
HBA1	**0**	**0**	**0**	**0**	**0**	**0**	**0**	**0**	**0**	**0**

Total clusters' TPM —TPM of representative consensus sequences that were computed for each of seven clusters

Total References sum TPM — total TPM of three already known HS2/HS3 sequences: X60726.1, S90110.1, X82942.1. The already known sequences were taken as references for quantification. In all the reassembled transcriptomes, they comprise 20-25% of all transcripts revealed.

ACTB —β-actin TPM. HBA1 — hemoglobin subunit alpha 1 TPM

Supplementary Table 2. HS2/HS3 TPM in GV and MII oocytes transcriptomes published by Reyes et al. (2017)

Donor age	Patient ID	HS2/HS3 clusters' TPM			
		GV (TPM)	MII (TPM)	GV (%)	MII (%)
40	AMA-1	10,79	8,86	100	82,14993
43	AMA-2	3,14	8,35	100	266,219
40	AMA-3	6,20	43,36	100	699,2973
42	AMA-4	2,44	6,58	100	269,5454
43	AMA-5	174,43	322,78	100	185,0472
27	YNG-1	8,53	75,38	100	883,7673
29	YNG-2	18,32	10,63	100	58,02074
29	YNG-3	2,17	12,68	100	585,5544
29	YNG-4	5,85	2,19	100	37,40918
20	YNG-5	16,95	84,72	100	499,7394

A

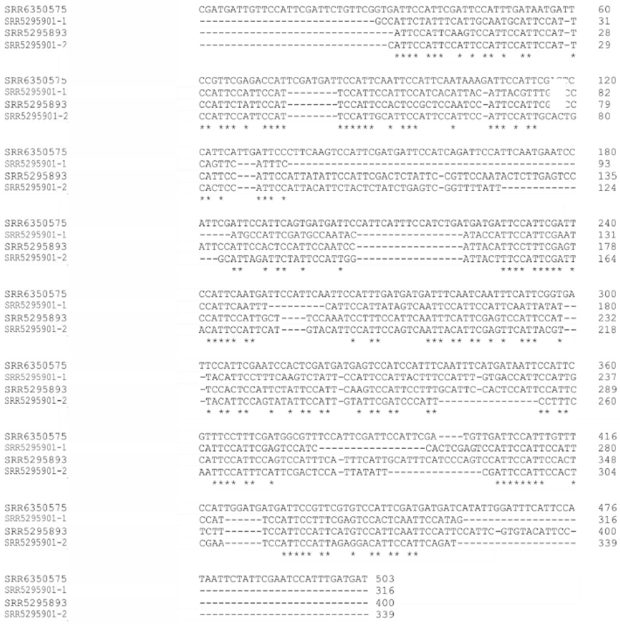

B

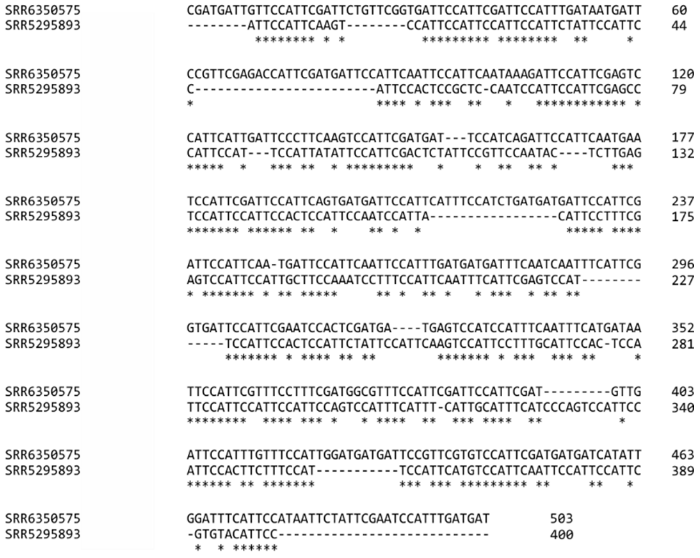

C

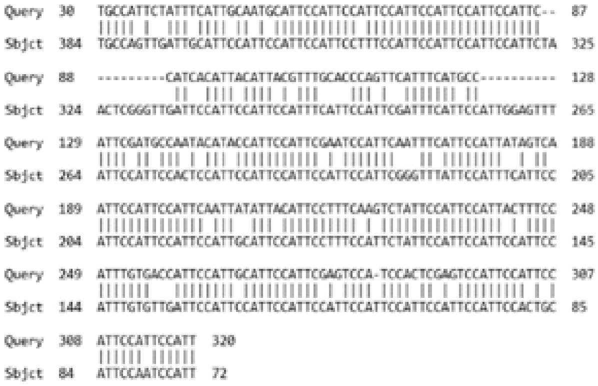

Supplementary Fig. S3. (A) Multiple sequence alignment of all four transcripts assembled from the specified SRR data of published human MI/MII oocyte transcriptomes. The transcripts were analyzed with CLUSTALO software. (B) Pairwise sequence alignment of the transcripts from two datasets, SRR6350575 and SRR5295893 (Supplementary Table 1), performed with the Emboss Needle software. (C) An example of alignment of an SRR5295901 HS transcript (Table 1) to a mRNA obtained previously from heat-shocked HeLa cells by Valgarsdottir et al. (2005). Query—the SRR5295901 Transcript 1 (Table 1 of the main text); subject—*Homo sapiens* clone 14 satellite III mRNA sequence Acc No AY845701.1 cloned by Valgarsdottir et al. (2005).

a.

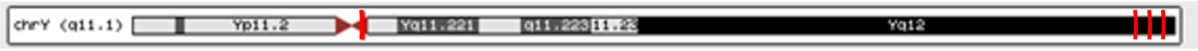

b.

	Description	Max Score	Total Score	Query Cover	E value	Per. Ident	Accession
☐	Homo sapiens chromosome 1, GRCh38.p12 Primary Assembly	87.8	94938	100%	3e-16	100.00%	NC_000001.11
☐	Homo sapiens chromosome 2, GRCh38.p12 Primary Assembly	87.8	2.132e+05	100%	3e-16	100.00%	NC_000002.12
☐	Homo sapiens chromosome 4, GRCh38.p12 Primary Assembly	87.8	6.457e+05	100%	3e-16	100.00%	NC_000004.12
☐	Homo sapiens chromosome 5, GRCh38.p12 Primary Assembly	87.8	60287	100%	3e-16	100.00%	NC_000005.10
☐	Homo sapiens chromosome 8, GRCh38.p12 Primary Assembly	87.8	2723	100%	3e-16	100.00%	NC_000008.11
☐	Homo sapiens chromosome 9, GRCh38.p12 Primary Assembly	87.8	8401	100%	3e-16	100.00%	NC_000009.12
☐	Homo sapiens chromosome 10, GRCh38.p12 Primary Assembly	87.8	1.747e+06	100%	3e-16	100.00%	NC_000010.11
☐	Homo sapiens chromosome 12, GRCh38.p12 Primary Assembly	87.8	6429	100%	3e-16	100.00%	NC_000012.12
☐	Homo sapiens chromosome 14, GRCh38.p12 Primary Assembly	87.8	46590	100%	3e-16	100.00%	NC_000014.9
☐	Homo sapiens chromosome 15, GRCh38.p12 Primary Assembly	87.8	19454	100%	3e-16	100.00%	NC_000015.10
☐	Homo sapiens chromosome 16, GRCh38.p12 Primary Assembly	87.8	94962	100%	3e-16	100.00%	NC_000016.10
☐	Homo sapiens chromosome 17, GRCh38.p12 Primary Assembly	87.8	1.472e+06	100%	3e-16	100.00%	NC_000017.11
☐	Homo sapiens chromosome 20, GRCh38.p12 Primary Assembly	87.8	9.320e+05	100%	3e-16	100.00%	NC_000020.11
☐	Homo sapiens chromosome 21, GRCh38.p12 Primary Assembly	87.8	7.937e+05	100%	3e-16	100.00%	NC_000021.9
☐	Homo sapiens chromosome 22, GRCh38.p12 Primary Assembly	87.8	2.613e+05	100%	3e-16	100.00%	NC_000022.11
☐	Homo sapiens chromosome Y, GRCh38.p12 Primary Assembly	87.8	2.183e+06	100%	3e-16	100.00%	NC_000024.10

Supplementary Fig. S4. The DYZ1 probe in the human genome. (a) Localization of the DYZ1 sequence (red lines) according to the UCSC Genome Browser program in the array of pericentromeric and long arm HS arrays of the Y chromosome (GRCh37/hg19 Assembly); (b) The representative fragment of DYZ1 alignment against the human reference genome (taxID: 9606) using BLAST software
